# Pragmatic Communication Deficit and Functional Outcome in Patients with Right- and Left-Brain Damage: A Pilot Study

**DOI:** 10.3390/brainsci14040387

**Published:** 2024-04-16

**Authors:** Simona Spaccavento, Sofia Caliendo, Roberta Galetta, Emilia Picciola, Ernesto Losavio, Robert Glueckauf

**Affiliations:** 1 Istituti Clinici Scientifici Maugeri—IRCCS, Institute of Bari, Via Generale Bellomo 73/75, 70124 Bari, Italy; sofia.caliendo@icsmaugeri.it (S.C.); roberta.galetta@icsmaugeri.it (R.G.); emilia.picciola@icsmaugeri.it (E.P.); ernesto.losavio@icsmaugeri.it (E.L.); 2 Department of Behavioral Sciences & Social Medicine, College of Medicine, Florida State University, 1115 W. Call St., Suite 4112, Tallahassee, FL 32306-4300, USA; robert.glueckauf@med.fsu.edu

**Keywords:** pragmatic deficits, stroke, functional outcome

## Abstract

Pragmatic communication abilities refer to the capacity to use language in a social context. Despite evidence to the contrary, the left cerebral hemisphere of the majority of right handers has been considered exclusively specialized for control of language phonology, syntax and semantics, whereas the right hemisphere has been specialized for the control of language pragmatics. Many studies have shown the non-exclusivity of the left hemisphere for language skills. Communication deficits observed in these studies for patients with right hemisphere damage confirmed the necessity for integrity of the right hemisphere across a number of language components. The aim of this study is to investigate the specific role of the right and left hemispheres across several aspects of communication deficits, with particular attention given to the influence of these deficits on functional outcome. The second aim is to characterize possible correlations between pragmatic and other cognitive deficits. We evaluated 22 patients, 15 with left- and 7 with right-brain ischemic or hemorrhagic damage, using cognitive, pragmatic and language tests. We deployed the Right Hemisphere Language Battery–Santa Lucia and Montreal d’Evaluation de la Communication to assess pragmatic abilities. The results showed no statistically significant differences between patients with left- and right-brain damage, highlighting the importance of integration between the two hemispheres in the communication process. Multiple significant correlations were found between pragmatic abilities and cognitive tests assessing global cognitive functioning, pantomime expression and comprehension. Pragmatic deficits were also shown to correlate with functional cognitive outcome. It is important to assess pragmatic abilities in patients with cognitive deficits after both left and right stroke for tailoring neuropsychological intervention to mitigate pragmatic disabilities in functional outcomes.

## 1. Introduction

Pragmatic communication abilities refer to the capacity to use language effectively in a social context [1]. Apart from verbal language and utterances, communication occurs through extra-linguistic means, such as gestures, body movements, and facial expressions. Thus, pragmatic communication abilities can be defined as the capacity to conduct communication interactions in a given social context, not only using language but also using contextual information processed via participants’ inferential abilities [2,3].

It is commonly believed that in the majority of right handers the left cerebral hemisphere is exclusively specialized for the control of language phonology, syntax and semantics, whereas the right hemisphere is specialized for the control of language pragmatics [4]. Many reports had shown the non-exclusivity of the left hemisphere for language skills. Communication deficits observed in these studies for patients with right hemisphere damage confirmed the necessity for integrity of the right hemisphere across a number of language components [5]. Patients with left hemispheric lesions and classical aphasic symptomatology are no longer the only patients showing communication problems.

Many studies have examined communication deficits of patients with right- and left-brain lesions [6]. Patients with right brain damage appear tangential, verbose, and inefficient in their expressive language [7]. They have also been shown to have difficulty comprehending nonliteral aspects of phrases, such as metaphor, proverbs, idioms and jokes [8]. In patients with left brain lesions, the findings of prior research have been inconsistent [9]. Some authors found a predominant role of the right hemisphere in pragmatic competence, whereas others reported left hemisphere involvement based on the specific pragmatic aspects they evaluated. It is important to note that the evaluation of pragmatic competence in patients with left brain injuries is tested through linguistic means, thus potentially influencing the results.

Note also that a right hemisphere lesion does not result in communication disorders in all individuals [10]. There are heterogeneous profiles of communication deficits in patients with right hemispheric lesions. These deficits could be influenced by other factors, such as lesion site and severity, age, education, pre-morbid communication profile and other concomitant cognitive deficits. However, there is a paucity of studies assessing the impact of these various factors on pragmatic deficits.

Accordingly, Gibbs et al. [11] noted that pragmatic and other linguistic components of language should be considered two sides of the same coin because linguistic components, such as syntactic or lexical aspects, must be integrated with communicative aspects, such as locus and time, to facilitate communication. Thus, pragmatics are not independent from language, but are a component of this cognitive function. Other authors [12] have found an association between communication abilities and other cognitive functions, such as working memory. Deficits in this ability can be affected by both linguistic and pragmatics skills.

The term aphasia should refer not only to more purely linguistic deficits, but also to pragmatic impairments. In some cases, the term pragmatic aphasia [13] is used to characterize pragmatic deficits following right hemispheric lesion. Recently, Minga et al. [14] proposed the term apragmatism as a potential diagnostic label for communication deficits after right hemispheric damage. This term denotes a specific set of disorders characterized by deficits in interpreting or conveying intended meaning through linguistic means, such as words and syntax or through prosody and emotions, and subsequently, via paralinguistic and extralinguistic means, such as gestures, facial expressions or body language.

Aphasia and apraxia are two deficits which often occur in association, due to the contiguity of brain area involved in both functions. This can be present despite reported cases of dissociations [15]. Apraxia is a higher-order motor disorder that causes an incapacity to make movements without sensorimotor deficit. These apraxic deficits occur more frequently after left hemisphere lesion (28–57%), with a prevalence from 0% to 34% in right-brain damaged patients [16]. Apraxic deficits can be differentiated into subtypes, according to the cognitive and anatomical system involved by the lesion. Rounis and Binkofski [17] have proposed a hierarchical model of apraxia, distinguishing between executive apraxia with deficits in execution of fine-motor hand skills and intermediate apraxia characterized by deficits in reaching and manipulating objects in the space and limb apraxia including pantomime, gesture imitation and comprehension.

Gestures are a particularly important aspect of the communication process. Limb apraxia is a movement disorder characterized by the inability to perform purposeful movements using tools or to make meaningful gestures which do not involve the use of objects. The pantomime is a mime of the use of a tool. According to the Roy’s model of apraxia [18], the integrity of the sensory/perceptual system and production system is not the only controlling set of factors; the pantomime depends on the patient’s knowledge of tools and actions and on the integrity of conceptual/semantic system. The correct use of tools is strongly influenced by the context, that is, with the same tool one can perform more pantomime productions. In addition, correct use of the tool may be influenced by examiner performance expectations. Thus, the correct pantomime depends on the knowledge of the social use of that specific tool.

Several studies [19,20,21] have reported a relationship between pantomime deficits and theory-of-mind deficits, which has characterized other neurological pathologies beside stroke and traumatic brain injury, such as autism spectrum disorders or schizophrenia. These pathologies have also manifested marked deficits in pragmatic and functional communications [22,23]. Moreover, gestures have an important role in communication, particularly in nonverbal communication. Producing a pantomime requires taking the perspective of the observer, such as is required in pragmatic communication. In this regard, there is limited research investigating the relationship between pragmatic deficits and pantomime in patients with left- and right-brain damage.

Despite the large impact of such deficits on daily life activities and global social participation, the effects of pragmatic communication deficits on functional outcome have been largely neglected in the literature. These deficits can have an important impact on social functioning, close interpersonal relationships, emotional adjustment and caregiver burden. In 2013, a new diagnostic category of Social Communication Disorders [24] was introduced in the fifth edition of the *Diagnostic and Statistical Manual (DSM-5)* [25], underscoring the importance of pragmatic abilities in communication. This disorder is characterized by a persistent deficit in the use of verbal and nonverbal communication in a social context, with important implications for social, scholastic and professional life. These difficulties can be observed in social interaction, social understanding, pragmatics and language processing. The assessment is based on interviews, observations, self-report questionnaires and measures completed by parents or other significant adults [24]. Hewetson et al. (2018) [26] have studied the communication disorders following right hemisphere lesions in everyday activities and found that patients with right lesions had more impairment in social participation and in occupational activities than healthy subjects.

The primary aim of this pilot study was to examine the role of right and left hemispheres across several aspects of communication deficits, with particular attention given to the influence of these deficits on functional outcome. The second aim is to characterize possible correlations between pragmatic and other cognitive deficits, pantomime execution, and comprehension.

## 2. Materials and Methods

### 2.1. Participants

Thirty-seven adults with vascular brain lesion (ischemic or hemorrhagic) consecutively admitted to the Neurorehabilitation Unit of Clinical Scientific Institutes “Maugeri” IRCCS—Bari (Italy) were enrolled in this study. Fifteen patients were excluded for bilateral or multiple lesions identified by neurodiagnostic scans. The study sample was composed of 22 persons with unilateral hemispheric lesions in the acute phase of disease (onset of stroke mean = 29.45 years), 15 with left brain damage and 7 with right lesion. The participants were all fluent monolingual Italian speakers and were hospitalized.

Inclusion criteria were first occurrence of a unilateral (ischemic or hemorrhagic) lesion, documented by computerized tomography or magnetic resonance imaging data; negative neurologic or psychiatric past history; and absence of cortical atrophy or leukoaraiosis. Bilateral lesions, previous stroke, non-cerebral involvement, or surgery (i.e., for aneurysm), and history of alcohol and drug abuse were exclusion criteria; patients with other chronic disabling pathologies (e.g., polyneuropathy, cancer, and limb amputation) or other central nervous system diseases were also excluded. None of the patients had hearing impairment; any visual deficits were corrected by wearing glasses.

Written consent was obtained from all family caregivers and participants in accordance with IRB regulations. The study was approved by the Ethics Clinical Scientific Institutes “Maugeri” committee (Prot. 47 CE). The study was conducted in accordance with the principles of the Declaration of Helsinki.

### 2.2. Materials

A complete neuropsychological battery was deployed to evaluate various cognitive domains as well as language and pragmatic deficits across all participants. First, a language evaluation was performed using the Aachener Aphasie Test (AAT) [27] to assess patients’ comprehension abilities. The AAT was administered by a speech and language therapist.

The following measures were administered by a neuropsychologist to all participants during the first week they arrived at the hospital. Note that patients were evaluated before starting any rehabilitation treatment. Two hours were required for the complete assessment. All patients were evaluated in the post-acute phase after their clinical conditions had stabilized.

#### 2.2.1. Language Assessment

Aachener Aphasie Test (AAT) [27] was used to assess linguistic abilities in both right- and left-brain damage. This battery is composed of six subtests: spontaneous speech, token test, repetition, written language, naming and comprehension.

#### 2.2.2. Cognitive Assessment

Global cognitive impairment was evaluated using the Mini-Mental State Examination (MMSE) [28,29]. This is a screening test for mental deterioration, assessing the following five areas: (a) orientation to time (score 0–5) and place (score 0–5); (b) immediate recall, i.e., short-term verbal memory (score 0–3); (c) attention and calculation (score 0–5); (d) delayed recall (score 0–3); (e) language section, with naming, repetition, comprehension, reading, and writing (score 0–6); and (f) constructional ability (score 0–3). The total score is 30. Scoring 24 or more on this test is considered diagnostic of normal cognitive status. The MMSE score must be corrected for age and education according to procedures standardized for the Italian population [30].

A more extended neuropsychological battery was used in order to investigate the cognitive functions, using the following tests:

Raven’s Colored Progressive Matrices [30,31,32,33] provides a non-verbal measure of intellectual ability. The patient must logically complete a given visual spatial pattern, choosing from a set of five alternatives.

- Phonological Verbal Fluency [30,31,32,33]: the patient is asked to produce as many words as possible beginning with the letters F, A and S in one minute.
- Semantic Verbal Fluency [34]: the patient is asked to say as many words as possible belonging to a given semantic category (colors, animals, fruits and cities) in two minutes.
- Attentive Matrices Test [34]: the test measures selective and sustained attention; the patient is asked to designate the target numbers from among the others.
- Copying Drawings [30,31,32,33]: the patient is required to copy three geometrical figures (star, cube and house).
- Copying Drawings with Landmarks [30,31,32,33]: this test uses the same figures as the previous test, but they are incomplete. The task involves completing the figure using landmarks already traced on paper.

#### 2.2.3. Buccofacial and Limb Praxic Evaluation

We used a battery composed of an ideomotor apraxia test, ideational apraxia, pantomime execution, pantomime comprehension and object-use test.

Ideomotor apraxia (IMA) was assessed by using a standard test [35,36] in which the task is to reproduce a wide variety of intransitive gestures, symbolic (e.g., sign of OK) or nonsymbolic (hand under the chin). The test includes 24 items with a score from 0 to 72. The diagnosis of IMA is reliable for scores under 53 points.

Ideational apraxia was assessed by evaluating the use of real objects (hammer, toothbrush, scissors, gun, pencil eraser, padlock and its key, candle, and a matchbox). The patient was asked to manipulate the object to demonstrate the correct use. A score of less than 14 was used to determine ideational apraxia.

For the recognition of pantomime, we used a pantomime recognition test, in which the patient was asked to indicate among 3 pictures the one that was correctly linked to the gesture. The total score is 20 (0–20).

We also evaluated the execution of objects pantomime through the pantomime-use objects test [35,36]. Test objects are presented to the patient who is asked to show the corresponding gesture without touching the object. The total score is 20 with a cut-off score of 18.

#### 2.2.4. Neglect Assessment

The possible presence of unilateral spatial neglect was evaluated through two cancellation tests:

1. Barrage test [37]: the patient has to cross out all lines (36) on the sheet. The score is based on the number of targets crossed out by the participant. The maximum score is 36 and the cut-off is 34.
2. Star cancellation test [38]: the subject is given an array containing 54 stars. The task is to cross out all the stars. The number of correctly crossed-out stars is computed, ranging from 0 to 54. The cut-off is 52.

#### 2.2.5. Pragmatic Assessment

For the evaluation of pragmatic abilities, we used two test batteries.

The Protocole Montréal d’Évaluation de la Communication (MEC)—Italian Version [39] provides a comprehensive examination of critical aspects of communication abilities including (a) comprehension and production of linguistic and emotional prosody, (b) lexical–semantic processes such as verbal fluency and semantic judgements, (c) conversational and narrative discourse, (d) pragmatic processes including the interpretation of indirect speech acts and metaphor, (e) self-awareness of deficits, and (f) informant awareness of deficits. In this study, we used only some subtests, such as self-awareness of deficit questionnaire, linguistic and emotional prosody, the interpretation of indirect speech acts, metaphors, and narrative discourse.

The Batteria sul Linguaggio dell’Emisfero Destro—(BLED) [40] consists of six subtests evaluating pictorial and written metaphors, inferences, requests, humor, and prosody. There is a score for each subtest (range 0–10).

#### 2.2.6. Functional Assessment

Functional Assessment Measure (FAM) Cognitive Subscale [41,42,43]: it assesses the individual’s level of independence, amount of assistance required, use of adaptive or assistive devises and the percentage of a given task completed successfully. This instrument includes 14 items assessing comprehension, expression, reading, writing, speech intelligibility, social interaction, emotional status, adjustment to limitation, employability, problem solving, memory, orientation, attention span and safety judgment. Each item is rated on a level of independence, with a score ranging from 1 “Total dependence” to 7 “Complete independence”. Scores are rated by multidisciplinary team members (i.e., a neuropsychologist, speech and language therapist, and a physiotherapist). Ratings are based on actual observed performance. In the data analysis, we examined the total and single-item scores, as well as the subtotal scores, such as linguistic (i.e., comprehension, expression, reading, writing, speech intelligibility), cognitive (i.e., problem-solving, memory, attention span, orientation), psychological (i.e., social interaction and emotional status) and awareness (i.e., adjustment to limitation and safety judgement).

## 3. Data Analysis

Descriptive statistics were calculated for demographic and medical characteristics of participants with right- and left-vascular brain lesions. Student’s *t* test, Chi-square and Mann–Whitney U were used to assess differences between the demographic and clinical data of the two groups. We computed the proportion of patients performing above and below the cut-off scores according to a single test’s normative data, and subsequently compared the frequencies between patients with right-hemispheric and left-hemispheric lesions using Chi-square tests.

Spearman’s correlation test was used to analyze the association between scores on pragmatic tests and mean scores obtained on the cognitive tests. *p* < 0.05 was considered statistically significant.

Data analyses were carried out using the Statistical Package for the Social Sciences (SPSS) Version 18.0 for Windows (IBM, Armonk, NY, USA).

## 4. Results

### 4.1. Demographic and Language Test Results

Table 1 shows the basic demographic and clinical features of the entire sample studied and those of the two groups. They were comparable for age, education and onset; a significant difference was found for gender. In regard to clinical features, significant differences between groups were found across most AAT subtests. As anticipated, patients with left brain damage performed worse on language tests, showing deficits exceeding those for spontaneous speech, repetition and writing. No differences were found for the AAT-Comprehension subtest, while a trend toward significance was evident for the AAT-Naming subtest.

### 4.2. Pragmatic Tests Results

Regarding the performance of patient groups on pragmatic test batteries (see Table 2), no significant differences were found on the BLED subtests and total scores. For the MEC scores (see Table 2), the two groups differed significantly only on the speech-act interpretation subtest; patients with left brain damage scored significantly lower than patients with right lesion on this measure.

### 4.3. Cognitive Test Results

For the cognitive tests (see Table 3), the results showed significant differences between two groups on MMSE score, Raven’s Progressive Matrices, and phonemic and semantic fluency. LBD patients showed poorer performance on these measures than their counterparts with right lesions, probably due to linguistic deficits.

### 4.4. Functional Outcome Results

Subsequently, we evaluated differences between functional outcomes at admission and at discharge for the entire sample and between the two groups (see Table 4); we also calculated the FAM gain score as the change in FAM total and subscores from admission to discharge. At admission, significant differences were found for the linguistic total FAM subscores, specifically for expression, reading and writing, with the LBD groups more impaired than RBD patients; at discharge, they were significantly different for cognitive and awareness subscores, with orientation and attention span, adjustment to limitation and safety judgment worse in RBD patients than in LBD. For gains differences, we found significant differences between the two groups in the following: in linguistic outcome, such as expression, reading, writing, and speech intelligibility; in cognitive outcome, as well as in orientation, problem-solving and attention-span subscales; in awareness outcome, such as adjustment to limitation and safety judgment; and in total functional outcome.

### 4.5. Correlations

The results of Spearman’s correlation analysis for the entire sample and the two groups showed significant correlations between a subset of cognitive tests and BLED and MEC subtests. For the MEC battery subscores, the MMSE score was positively associated with narrative discourse partial (rho = 0.51 *p* < 0.05) and total (rho = 0.7 *p* < 0.01) retelling. The attentive matrices score was significantly associated with the speech-act interpretation subscore (rho = 0.43 *p* < 0.05). We found significant correlations between the praxic evaluation tests with the emotional prosody comprehension subtest: in particular, significant positive correlations were found with the buccofacial apraxia test (rho = 0.52 *p* < 0.05), pantomime comprehension test (rho = 0.54 *p* < 0.05) and expression pantomime test rho = 0.47 (*p* < 0.05). For the BLED battery, Raven’s Colored Test score was positively correlated with the comprehension of pictures subtest (rho = 0.5 *p* < 0.05), and with comprehension of indirect requests (rho = 0.47 *p* < 0.05). Pantomime comprehension and expression tests were associated with the following: the first with the comprehension of inferences subtest (rho = 0.44 *p* < 0.05), the comprehension of indirect requests subtest (rho = 0.55 *p* < 0.01), and the BLED total score (rho = 0.52 *p* < 0.05), and the second with the comprehension of humoristic expression subtest (rho = 0.58 *p* < 0.01) and the BLED total score (rho = 0.48 *p* < 0.05).

We also calculated the Spearman’s correlations for the two groups separately. In the LBD group, we found positive correlations between MMSE and BLED comprehension of written metaphors (rho = 0.65 *p* < 0.05), BLED comprehension of indirect requests (rho = 0.62 *p* < 0.05) and BLED total score (rho = 0.62 *p* < 0.05); moreover, the pantomime expression test correlated with the comprehension of humoristic expressions (rho = 0.57 *p* < 0.05) and the BLED total score (rho = 0.56 *p* < 0.05). Analyzing the correlations between MEC subtests and cognitive measures, we found between MMSE and narrative discourse partial retelling (rho = 0.71 *p* < 0.01) and total retelling (rho = 0.9 *p* < 0.001). These MEC subtests correlated also with the pantomime expression test (respectively, rho = 0.6 *p* < 0.05 and rho = 0.62 *p* < 0.01). Emotional prosody comprehension was positive correlated with the buccofacial apraxia test (rho = 0.59 *p* < 0.05) and with pantomime expression (rho = 0.53 *p* < 0.05).

In the group of patients with RBD, we found high correlations between the BLED prosody subtest and the ideomotor apraxia test (both the significant and non-significant test: rho = 0.77 *p* < 0.05; rho = 0.77 *p* < 0.05), between the BLED comprehension of indirect requests and pantomime comprehension (rho = 0.82, *p* < 0.05) and between Raven’s Matrices and the BLED comprehension of written metaphors (rho = 0.76, *p* < 0.05) and BLED comprehension of indirect request (rho = 0.78 *p* < 0.05). For the MEC protocol, high correlation coefficients were found between the pantomime comprehension test and linguistic prosody–comprehension (rho = 0.94 *p* < 0.01), emotional prosody–comprehension (rho = 0.8, *p* < 0.05) and emotional prosody–repetition (rho = 0.91, *p* < 0.01). Also, Raven’ Matrice test score correlated positively with the same MEC subtests (respectively, rho = 0.93 *p* < 0.01, rho = 0.82 *p* < 0.05, and rho = 0.8, *p* < 0.05), more than with the speech-act interpretation subtest (rho = 0.81 *p* < 0.05).

## 5. Discussion

The current pilot study examined the linguistic and pragmatic abilities of patients with right and left brain lesions, focusing on associations between pragmatic and cognitive deficits, as well as the influence of pragmatic deficits on cognitive functional abilities. The use of two pragmatic test batteries facilitated assessment of pragmatic competencies and the role of both hemispheres in each function.

The results showed no substantial differences in the overall performance of the subtests between the two groups. The only significant difference between patients with right- and left-hemispheric damage occurred in the MEC speech-act interpretation subtest, on which the former participants performed significantly better than the latter. This was probably attributable to the linguistic component of this subtest, which was impaired in patients with left brain lesions and aphasia.

The left hemisphere has been traditionally considered to have a dominant role for the rule-based components of language. A growing number of studies have substantiated the involvement of the left hemisphere in pragmatic competence. Comparing the pragmatic abilities of right- and left-hemisphere-damaged patients, Cutica et al. [44] showed greater impairment in patients with left brain damage than controls with right brain damage on complex non-standard acts. In this pilot study, the patients with left hemispheric lesions showed greater impairment in more complex indirect pragmatic tasks (e.g., inference and humor) than simple ones (e.g., requests). In patients with right brain damage, no significant differences on these tasks were obtained. Cutica et al. [44] found that pragmatic performance is better preserved in patients with left hemisphere damage than those with right hemisphere damage. Comparing the two groups’ performance on extralinguistic tasks with healthy controls, the authors found that both patients with right- and left-brain damage performed significantly worse than controls, further buttressing the involvement of both hemispheres in the comprehension of communication interactions. Patients with right hemisphere damage also showed more difficulties in the easiest kind of pragmatic tasks.

Note that Soroker et al. [45] also reported the involvement of both right and left hemispheres for pragmatic competence. In their evaluation of the processing of basic speech acts, such as question, assertion, request and command, patients with left hemispheric lesions performed worse than patients with right hemispheric lesions.

In a study examining the differences in pragmatic deficits between patients with left- and right-brain damage, Zaidel et al. [46] evaluated the pragmatic abilities of patients with left- and right-brain lesions using the Right Hemisphere Communication Battery (RHCB). It includes 11 subtests: pictoral humor, verbal humor, humor production, prosody, indirect requests, pictoral metaphors, verbal metaphors, inferences, sarcasm, alternative word meanings, and narrative comprehension. In a sample of 27 patients with right brain damage, 31 with left brain damage and 21 healthy controls, the authors found that both patient groups were significantly impaired compared to controls on most subtests, such as humor, sarcasm, and prosody, confirming the involvement of both hemispheres in these communication domains. In contrast, the only differences between two patient groups were found in indirect requests and verbal metaphors subtests, with more impairment of patients with left brain lesion. According to the authors, the results suggested involvement of both hemispheres in pragmatic functions.

An important aspect of our study is the relationship found between pragmatic test performances and neuropsychological test scores for both the entire sample and the two separate groups. Particularly noteworthy is the fact that the correlation between the prosody and pantomime tests has not been reported in previous research. Most studies assessing the association between pragmatic deficits and other cognitive impairments had proposed a linkage with executive dysfunction [47,48,49]. In a sample of 30 patients with damage of the prefrontal cortex, Ouerchefani et al. [50] found significant associations among impairment of pragmatic abilities, deficits in executive functions, and theory-of-mind (ToM) tests. Tsolakopoulos et al. [51] found frequent co-occurrences of executive deficits, ToM deficits and pragmatic impairments in patients with right hemispheric stroke, suggesting that pragmatic deficits could stem from deficits in executive and ToM cognitive abilities. The pragmatic functions would have a social–cognitive component, as well as a linguistic–communication component.

This hypothesized relationship was supported in our pilot study’s pattern of correlations between prosody and pantomime tests, which have not been reported previously in the literature to our knowledge. Communication intentions can be expressed using the linguistic modality, such as language, but also through extralinguistic and non-verbal modalities, such as gestures. The latter can have both communication value and social implications. Contrary to our pilot study group with left brain damage, significant positive correlations were found between pantomime comprehension and linguistic and emotional prosody in the group with left brain damage. For the latter, the ability to recognize gestures related to tools was significantly correlated with the ability to recognize linguistic prosody and to repeat emotional sentences after recognizing them. Furthermore, patients with right lesions consistently showed significant positive correlations with Raven’s Colored Matrices Test scores and non-standard communication task scores, such as metaphors and comprehension of indirect requests.

The relationship among these cognitive elements could be explained by the important role of the dorsolateral prefrontal cortex, as many studies demonstrated [52]. Beside language and speech processing and functions, such as discourse management, prosody, comprehension of nonliteral language, and inferences, this area is implicated also in nonlinguistic functions, such as executive control, working memory, theory of mind, and mood regulation.

Only a small number of studies [26,53,54] have delineated the negative impact that people with cognitive communication deficits after right hemispherical lesion experience during social interactions in daily activities. The scarcity of studies may be attributable to the important and predominant role that unilateral spatial neglect has on the functional outcome of patients with right brain damage, but could also be due to the low sensitivity level of acute neurological scales in identifying people with communication deficits after right hemispheric lesion. In a retrospective study, Hewetson et al. [26] found a comparable frequency between aphasia (68%) and cognitive–communication deficits (66%) after right lesion; moreover, there were no significant differences in functional gains between the two groups. In our study, the two groups were significantly different on the FAM linguistic subscale, expression, reading and writing items at hospital admission and for the cognitive subscale, on orientation and attention-span items at discharge. The gain scores of the two groups were significantly different, not only for linguistic outcome, but also for cognitive subscores (i.e., problem-solving, orientation and attention span) and awareness (i.e., safety judgement and adjustment to limitation). To our knowledge, no previous studies have investigated the specific role of pragmatic abilities on functional cognitive outcome. Based on our pilot study findings, speech-act interpretation appears important in shaping the initial functional outcomes following left hemispheric stroke.

A major limitation of this study was small sample size. Future studies with larger samples are needed to test the reliability of the current findings. A second limitation was lack of a healthy control group with which to compare the performance of brain-damaged patients.

In addition to replicating the study with a larger sample, a promising direction for future research is to assess the relationships among gestures, pantomime with prosody, and empathy abilities, using measures with high ecological validity. It is important to evaluate the role of theory of mind and the correlation with some specific pragmatic abilities. The involvement in the study of patients with left hemispherical damage and then with language deficits makes it necessary to use well-selected tests to bypass the possible language comprehension deficits.

Furthermore, the use of contextually-specific outcome measures, such as the Functional Outcome Questionnaire for Aphasia (FOQ-A) [55] or the Communicative Effectiveness Index (CETI) [56], would facilitate evaluation of the impact of pragmatic deficits on everyday social and communicative situations. Future research should also examine the role of specific rehabilitation training on the improvement of pragmatic abilities in stroke samples, both with left- and right-brain damage.

## 6. Conclusions

The findings of the pilot study underscored the important role of the right and left hemispheres in pragmatic communication. Strong associations were found between pragmatic test performances and cognitive tests, thus attesting to the significant influence of both hemispheres on the efficacy of communication. Having a clear understanding of the contributions of both hemispheres on pragmatic communication is likely to facilitate effective tailoring of rehabilitation treatment and, in turn, increase the likelihood of functional improvement in persons with brain injury.

## Figures and Tables

**Table 1:** Demographic and clinical features of patients.

	All Patients (N = 22)	LBD Patients (N = 15)	RBD Patients (N = 7)	Comparison between Two Groups
Demographic features				
Age (years) (mean ± SD)	62 ± 12.1	60.47 ± 12.94	65.29 ± 10.16	t_(20)_ = 0.87 *p* = 0.4
Education (years) (mean ± SD)	9.86 ± 3.45	10.07 ± 3.9	9.4 ± 2.44	t_(20)_ = −0.39 *p* = 0.69
Gender (M/F) (%)	16/6 (72.7/27.3)	13/2 (86.7/13.3)	3/4 (42.9/57.1)	X^2^ = 4.55 df = 1 *p* = 0.03
Clinical features				
Aphasia (%)	10/22 (45.4)	10/15 (66.6)	0/7 (0)	X^2^ =0.18 df = 1 *p* = 0.67
Neglect (%)	4/22 (18.2)	0/15 (0)	4/7 (57.1)	X^2^ =8.91 df = 1 *p* = 0.003
Onset (months) (mean ± SD)	29.45 ± 17.15	25.6 ± 15.95	37.71 ± 17.888	t_(20)_= 1.6 *p* = 0.13
AAT-Communicative Behaviour	3.77 ± 1.23	3.2 ± 1.08	5 ± 0.00	U = 7 *p* = 0.001
AAT-Articulation and Prosody	4.18 ± 1.1	4 ± 1.25	4.57 ± 0.54	U = 43 *p* = 0.461
AAT-Automatized Language	4.41 ± 1.01	4.13 ± 1.13	5 ± 0.00	U = 28 *p* = 0.036
AAT-Semantic Structure	4.09 ± 1.06	3.73 ± 1.1	4.86 ± 0.38	U = 19 *p* = 0.011
AAT-Phonemic Structure	4.09 ± 1.15	3.67 ± 1.18	5 ± 0.00	U = 17.5 *p* = 0.007
AAT-Syntactic Structure	4.00 ± 1.19	3.6 ± 1.24	4.86 ± 0.38	U = 21.5 *p* = 0.019
AAT-Token Test	10.82 ± 9.41	13.53 ± 10.16	5 ± 3.37	U = 24 *p* = 0.044
AAT-Repetition	132.27 ± 24.45	125.67 ± 27.32	146.43 ± 2.82	U = 22 *p* = 0.031
AAT-Writing	75 ± 19.06	69 ± 20.45	87.86 ± 3.53	U = 13.5 *p* = 0.006
AAT-Naming	99.82 ± 23.87	93.07 ± 26.13	114.29 ± 6.5	U = 25.5 *p* = 0.056
AAT-Comprehension	102.5 ± 11.13	102.4 ± 8.87	102.71 ± 15.8	U = 45.5 *p* = 0.621

AAT = Aachener Aphasie Test; F = female; M = male; LBD = Left Brain Damaged; RBD = Right Brain Damaged.

**Table 2:** BLED and MeC total subtest scores of all patients and of two groups.

	All Patients (N = 22)	LBD Patients (N = 15)	RBD Patients (N = 7)	Comparison between Two Groups
BLED Scores				
Comprehension of pictures (mean ± SD)	5.18 ± 2.54	5.6 ± 2.17	4.29 ± 3.2	U = 37.5 *p* = 0.28
Comprehension of written metaphors	7.64 ± 2.46	7.53 ± 2.83	7.86 ± 1.57	U = 49 *p* = 0.8
Comprehension of inferences	5.39 ± 1.28	5.23 ± 1.19	5.71 ± 1.5	U = 47.5 *p* = 0.72
Comprehension of indirect requests	8.05 ± 1.94	8.33 ± 1.78	7.43 ± 2.44	U = 41 *p* = 0.41
Comprehension of humoristic expressions	5.27 ± 2.31	5.13 ± 2.7	5.57 ± 1.27	U = 50.5 *p* = 0.89
Prosody	6.5 ± 1.95	5.13 ± 2.7	7.14 ± 1.86	U = 40.5 *p* = 0.39
Total score	37.43 ± 8.15	37.57 ± 7.35	37.14 ± 10.31	U = 52 *p* = 0.97
MEC Protocol Scores				
Questionnaire on deficit awareness	4.63 ± 1.67	4.31 ± 1.75	5.33 ± 1.37	U = 26 *p* = 0.24
Metaphor comprehension	31.91 ± 7.57	30.13 ± 7.65	35.71 ± 6.26	U = 32 *p* = 0.14
Speech-act interpretation	33.32 ± 5.98	31.4 ± 6.16	37.43 ± 2.76	U = 21.5 *p* = 0.03
Linguistic prosody—Comprehension	9.45 ± 2.92	9.53 ± 2.92	9.29 ± 3.15	U = 52.5 *p* = 1
Linguistic prosody—Repetition	10.55 ± 2.84	10.27 ± 3.35	11.14 ± 1.22	U = 51.5 *p* = 0.94
Emotional prosody—Comprehension	8.27 ± 2.51	8.27 ± 2.6	8.29 ± 2.5	U = 50 *p* = 0.89
Emotional prosody—Repetition	6.64 ± 4.45	6.4 ± 4.72	7.14 ± 4.1	U = 48 *p* = 0.75
Emotional prosody—Production	10.82 ± 6.05	10.67 ± 5.8	11.14 ± 7	U = 48 *p* = 0.75
Narrative discourse				
Partial re-telling	12.91 ± 6.56	11.4 ± 6.2	16.14 ± 5.52	U = 31 *p* = 0.13
Total re-telling	7.32 ± 3.77	6.27 ± 3.9	9.57 ± 1.98	U = 29.5 *p* = 0.1
Comprehension questions	7.64 ± 3.37	7.47 ± 3.74	8 ± 2.65	U = 50.5 *p* = 0.89

BLED = Batteria sul Linguaggio dell’Emisfero Destro; MEC = Protocole Montréal d’Évaluation de la Communication; LBD = Left Brain Damaged; RBD = Right Brain Damaged.

**Table 3:** Cognitive test scores of all patients and of two groups.

	All Patients (N = 22)	LBD Patients (N = 15)	RBD Patients (N = 7)	Comparison between Two Groups
MMSE	23.64 ± 3.61	22.02 ± 3.46	26.61 ± 1.2	U = 5.5 *p* = 0.006
Raven’s Colored Progressive Matrices Test	22.59 ± 5.88	5.49 ± 5.24	18.76 ± 24.51	U = 22 *p* = 0.04
Phonemic Fluency	12.52 ± 10.22	7.93 ± 8.98	21.04 ± 6.24	U = 10 *p* = 0.005
Semantic Fluency	8.66 ± 5.1	6.67 ± 4.43	12.36 ± 4.258	U = 14 *p* = 0.01
Attentive Matrices	31.44 ± 15.03	27.87 ± 2.44	38.14 ± 17.13	U = 29 *p* = 0.09
Copying Drawings	8.4 ± 1.55	8.72 ± 1.43	7.73 ± 1.68	U = 32 *p* = 0.15
Copying Drawings with landmarks	62.6 ± 9.54	64.31 ± 7.97	58.96 ± 12.15	U = 29 *p* = 0.09
BuccoFacial Apraxia	18.56 ± 2.2	18.15 ± 2.5	19.43 ± 0.98	U = 41.5 *p* = 0.37
Ideomotor Apraxia—Significative gestures	29 ± 1.93	28.8 ± 2.21	29.43 ± 1.13	U = 50 *p* = 0.82
Ideomotor apraxia—NonSignificative Gestures	28.73 ± 2.12	28.93 ± 1.67	28.29 ± 2.98	U = 50.5 *p* = 0.86
Pantomime Comprehension	18.64 ± 1.97	18.73 ± 2.22	18.43 ± 1.39	U = 37.5 *p* = 0.25
Pantomime Expression	19.27 ± 1.58	19.07 ± 1.83	19.71 ± 0.76	U = 45 *p* = 0.47
Ideational Apraxia	15.91 ± 0.43	15.87 ± 0.52	16 ± 0.00	U = 49 *p* = 0.49
Line’s Barrage	34.8 ± 2.39	35.67 ± 0.58	34.43 ± 2.82	U = 10 *p* = 0.89
Star’s Barrage	45.2 ± 14.52	48 ± 9.54	44 ± 16.74	U = 9.5 *p* = 0.81

MMSE = Mini-Mental State Examination; LBD = Left Brain Damaeg; RBD = Right Brain Damaged.

**Table 4:** FAM Cognitive subscale scores at admission and at discharge, and the FAM gains.

	Admission			Discharge			Gain		
Test	LBD	RBD		LBD	RBD		LBD	RBD	
FAM									
Comprehension	4.2 ± 1.9	5.86 ± 1.21	*p* = 0.05	5.4 ± 1.45	6.28 ± 0.95	*p* = 1.17	1.28 ± 0.91	0.6 ± 0.55	*p* = 0.13
Expression	3.6 ± 1.99	6 ± 1.15	*p* = 0.01	5 ± 1.46	6.14 ± 0.89	*p* = 0.08	1.4 ± 0.99	0.14 ± 0.38	*p* = 0.005
Reading	3.66 ± 1.88	5.43 ± 1.51	*p* = 0.04	5.07 ± 1.44	5.57 ± 1.51	*p* = 0.45	1.4 ± 1.18	0.14 ± 0.38	*p* = 0.006
Writing	3.6 ± 1.99	5.57 ± 1.4	*p* = 0.03	4.93 ± 1.49	5.57 ± 1.51	*p* = 0.42	1.3 ± 1.29	0.0 ± 0.58	*p* = 0.004
Speech Intelligibility	4.4 ± 1.8	5.71 ± 1.25	*p* = 0.09	5.87 ± 1.19	5.86 ± 1.07	*p* = 0.88	1.47 ± 1.24	5.86 ± 15.05	*p* = 0.01
Linguistic FAM	19.47 ± 8.98	28.57 ± 5.99	*p* = 0.03	26.27 ± 6.43	35.14 ± 14.08	*p* = 0.32	6.8 ± 5	0.86 ± 1.21	*p* = 0.004
Social Interaction	4.4 ± 1.8	4.86 ± 1.46	*p* = 0.54	5.53 ± 1.19	5.43 ± 1.27	*p* = 0.8	1.13 ± 0.99	0.57 ± 1.51	*p* = 0.38
Emotional Status	3.6 ± 1.59	3.57 ± 1.51	*p* = 0.88	5 ± 1.13	3.57 ± 1.72	*p* = 0.07	1.4 ± 1.12	0.0 ± 1.41	*p* = 0.36
Psychological FAM	8 ± 3.18	8.43 ± 2.7	*p* = 0.77	10.53 ± 2.13	9 ± 2.7	*p* = 0.19	2.53 ± 1.85	0.57 ± 2.57	*p* = 0.13
Adjustment to limitation	3.4 ± 1.59	3.86 ± 1.17	*p* = 0.51	5 ± 1.36	3.14 ± 1.57	*p* = 0.02	1.6 ± 1.35	0.28 ± 0.75	*p* = 0.02
Employability	2.53 ± 1.3	1.86 ± 0.69	*p* = 0.23	3.4 ± 1.88	2 ± 0.82	*p* = 0.09	0.87 ± 1.1	0.14 ± 0.69	*p* = 0.13
Problem-solving	3.2 ± 1.42	2.86 ± 1.46	*p* = 0.58	4.8 ± 1.26	3.29 ± 1.6	*p* = 0.07	1.6 ± 1.18	0.43 ± 0.97	*p* = 0.04
Memory	3.8 ± 1.61	3.57 ± 1.62	*p* = 0.72	5 ± 1.3	4.28 ± 1.5	*p* = 0.31	1.2 ± 1.15	0.71 ± 0.76	*p* = 0.39
Orientation	4.2 ± 1.82	4.43 ± 1.13	*p* = 0.61	6.06 ± 1.09	4.43 ± 1.4	*p* = 0.01	1.87 ± 1.55	0.0 ± 1	*p* = 0.008
Attention span	4.1 ± 1.62	3.57 ± 0.79	*p* = 0.56	5.6 ± 1.05	4 ± 1	*p* = 0.01	1.53 ± 1.1	0.43 ± 1.13	*p* = 0.04
Cognitive FAM	15.27 ± 6.18	14.43 ± 4.12	*p* = 0.97	21.47 ± 4.29	16 ± 4.83	*p* = 0.02	6.2 ± 3.98	1.57 ± 3.41	*p* = 0.007
Safety Judgement	3.53 ± 1.6	2.86 ± 0.9	*p* = 0.44	5.27 ± 1.28	3.14 ± 1.07	*p* = 0.01	1.7 ± 1.16	0.28 ± 0.49	*p* = 0.02
Awareness FAM	6.9 ± 2.87	5.71 ± 1.7	*p* = 0.41	10.27 ± 2.46	6.29 ± 2.43	*p* = 0.01	3.33 ± 2.13	0.57 ± 0.98	*p* = 0.008
Total score	52.2 ± 20.07	58.14 ± 14.29	*p* = 0.41	70.93 ± 15.65	61.14 ± 15.88	*p* = 0.18	19.43 ± 9.85	1.8 ± 7.98	*p* = 0.005

FAM = Functional Assessment Measure; LBD = Left Brain Damaged; RBD = Right Brain Damaged.

## Data Availability

The data presented in this study are available on request from the corresponding author. The data are not publicly available due to specific ethical and privacy considerations.
